# The Acceptability, Safety and Impact of a Play Co‐Developed With Public Contributors as a Format for Disseminating Research on a Sensitive Subject

**DOI:** 10.1111/hex.70074

**Published:** 2024-11-06

**Authors:** Cat Papastavrou Brooks, Noreen Hopewell‐Kelly, Natalia V. Lewis

**Affiliations:** ^1^ Bristol Medical School, Centre for Academic Primary Care, Population Health Sciences University of Bristol Bristol UK; ^2^ NIHR Bristol Biomedical Research Centre University Hospitals Bristol and Weston NHS Foundation Trust and University of Bristol Bristol UK; ^3^ Marie Curie Palliative Care Research Centre Cardiff University Cardiff UK

**Keywords:** co‐production, creative methods, domestic abuse, domestic violence, Patient and Public Involvement, PTSD, research dissemination, theatre

## Abstract

**Introduction:**

Patient and public involvement (PPI) and dissemination of research findings are key parts of the pathway to research impact; however, traditional approaches often fail to engage non‐academic audiences. Creative methods such as co‐developed plays can be effective ways of making the research process and findings more engaging and accessible to the public. Not much is known about how to safely involve patients and the public in the development and delivery of plays disseminating research on sensitive subjects. Members of a PPI group on a study about mindfulness for women with a history of domestic abuse co‐developed and performed a play about their experiences. This study aimed to evaluate the impact, acceptability and safety of a co‐developed play in publicizing PPI and findings from research on domestic abuse.

**Methods:**

We conducted a mixed‐methods study with the play team and audience. We collected 20 quantitative and 56 qualitative survey responses from audience members, carried out 4.25 h of direct observations of play performances and interviewed seven audience members and eight play team members. Data were analyzed using the framework method and descriptive statistics, using a ‘following a thread’ approach to integrate qualitative and quantitative findings in themes answering our study aim.

**Findings:**

We developed three integrated themes with ten sub‐themes. The ‘Value’ theme summarized the plays' impact on audience understanding, potential mechanisms of impact and its effectiveness in depth over breadth of dissemination. The ‘Re‐traumatization’ theme described potential harms of the play, the risks of re‐traumatizing actors and distressing audiences. The ‘Reducing the risks’ theme summarized ways of reducing these risks of harm.

**Conclusion:**

A play co‐developed and performed by study PPI members raised awareness of domestic abuse. However, there were divergent opinions on its value in disseminating messages about PPI in research on sensitive subjects. The value of the play for research dissemination was linked to its ability to emotionally engage the public, and to its accessibility. Implementing strategies to reduce the risk of re‐traumatizing audience members and the project team is recommended.

**Patient or Public Contribution:**

Everyone with direct experience of co‐creating and performing the play contributed to this study. This included four public contributors: a community theatre producer, two actors with lived experience of domestic abuse who were members of the study PPI group and one community actor already working with the community theatre. A participatory workshop with PPI contributors was held to refine our research questions and data collection instruments, using a public involvement evaluation tool, The Cube. PPI contributors checked and commented on the draft manuscript.

## 1 Introduction

### 1.1 Background

Effective research dissemination involves maximizing the benefits of research by transmitting research findings in a timely manner to those who can make use of them [1]. Although most researchers are committed to disseminating to non‐academic audiences, dissemination beyond research publications often occurs in an ad hoc fashion [2], with an overreliance on passive diffusion as opposed to active dissemination [3]. This can lead to a ‘gap’ between academic research and the ‘end‐users’ of research – the general public, patients and carers [3, 4]. A lack of community involvement in dissemination activities can lead to decreased engagement in research [5] and an erosion of trust in researchers [6]. Empirical work in this area suggests that although funding requirements and perceived funder priorities play a key role in issues with research dissemination to general audiences [7, 8], a lack of knowledge, guidance and evaluation metrics around dissemination are also important contributors [7, 8, 9].

Theatre forms part of a cluster of art‐based dissemination approaches that have established effectiveness at disseminating health research to non‐academic audiences through increasing accessibility and engagement with findings [10, 11, 12, 13]. Collaborative theatre‐based methods often utilize innovative co‐production approaches in creating dissemination outputs [14]. Previous work has evaluated the impact of researchers and theatre‐makers co‐creating plays with men with prostate cancer [15], women with breast cancer and medical oncologists [16], communities with a high prevalence of glaucoma [17] and sexual and gender minorities accessing fertility services [18]. There are also examples of theatre as a dissemination format for health research not involving a co‐development process with stakeholders [19, 20, 21]. These theatre‐based forms of dissemination have an established impact on knowledge and awareness of health conditions [17, 18, 19, 20] through enabling audiences to emotionally connect to the experience of patients suffering from them [15, 16].

Although using plays for disseminating research across multiple disease areas could be re‐traumatizing to both audience members and those co‐creating it, limited research exists on strategies to minimize this risk. This is despite one study reporting that the audience had found the play on experiences of cancer treatment particularly emotionally difficult [15]. A trauma‐informed approach can be a safer way to involve patients and the public in disseminating findings from studies on sensitive topics. Guidance on the trauma‐informed approach emphasizes the core principles of safety, trustworthiness and transparency, peer support, collaboration and mutuality, empowerment and choice and cultural, historical and gender issues [22]. These principles have been applied to trauma‐informed co‐production within health research, with a particular focus on ensuring the safety, trust and empowerment of contributors during co‐production [23, 24, 25]. The process of creating original theatre, especially if it is based on personal experiences of illness, distress or traumatic experiences, could have the potential to be re‐traumatizing or triggering to contributors in differing ways from standard co‐production processes, as it can involve re‐enacting and spending prolonged time revisiting traumatic experiences as part of play development [26, 27]. This suggests that specific adaptations may be needed on co‐producing theatre‐based dissemination in a trauma‐informed way, beyond the general guidance for research co‐production [28].

### 1.2 The ‘Hard Evidence’ Play

In December 2020, we secured a grant to explore the value of Patient and Public Involvement (PPI) in disseminating research on a sensitive topic. The project was built on the PPI component of a feasibility study of a trauma‐specific mindfulness intervention for survivors of domestic abuse, the coMforT study [29]. Supported by the grant, two out of the six members from the study PPI group (who were survivors of domestic abuse), the PPI lead and the director of a community theatre co‐developed a play, ‘Hard Evidence’. This play was based on the experiences of the coMforT PPI members and aimed to disseminate information about the value of PPI in publicizing research on sensitive subjects and to raise awareness about research on domestic abuse. The play follows Jan, a survivor of domestic abuse who has been a PPI contributor on a research project, and her friend Christine. When Jan recognizes that Christine's relationship shows signs of domestic abuse, she draws on the newfound confidence that she has gained through using her lived experience as a PPI contributor to support her friend.

Due to Covid restrictions, the initial development of ‘Hard Evidence’ took place online. The public contributors and the director of the community theatre co‐wrote the story line and script, which was then rehearsed in weekly online meetings. The coMforT PPI lead remained involved in the ongoing work by reviewing the script and attending rehearsals ad hoc. The lead coMforT researcher reviewed the script and provided feedback to the director.

The play was filmed at a closed ‘in‐person’ performance in April 2021 without an audience and disseminated through social media (239 views as of 03 April 2024). A live performance was possible post Covid and funding was secured to restart rehearsals. The groups' members met in person, weekly at the community theatre. Rehearsals lasted for 7 months, with two public performances in November 2021 at the theatre base and two performances in July 2022 in community venues. Each play was followed by a Q&A session. In total, the play was attended by 156 people, with 80 and 42 people attending the first two performances and 8 and 26 attending the performances in community venues.

### 1.3 Research Objectives

This study aimed to evaluate the process of co‐producing and performing the ‘Hard Evidence’ play and its impact on raising awareness about domestic abuse and PPI in research on a sensitive topic. The evaluation of impact assessed to what extent the play changed individuals' knowledge about domestic abuse and PPI in research. The process evaluation explored the acceptability and safety of using theatre for disseminating research findings and publicizing PPI in research on a sensitive topic.

## 2 Materials and Methods

### 2.1 Design

The study was conducted by a team of two health services researchers and a PPI lead with a research background based at a university in the Southwest of England, UK. We aimed to adhere to the principles of co‐production: sharing power, including all perspectives and skills, respecting and valuing the knowledge of everyone, reciprocity and mutuality, building and maintaining relationships [30]. This evaluation study consisted of two consecutive phases.

### 2.2 Phase 1. Co‐Development of Evaluation Project

We organized a 3‐h participatory workshop in a community centre. The workshop aimed to refine our research questions and data collection instruments. The first author invited individuals with direct experience of co‐producing and performing the play. An external PPI coordinator facilitated the workshop. Participants manually completed the public involvement evaluation tool The Cube on wall charts [31] and discussed their experiences of creating and performing the play. The first author took fieldnotes, manually categorized workshop artefacts and fieldnotes by themes and amended study research questions, protocol and documentation to incorporate the proposed changes.

### 2.3 Phase 2. ‘Hard Evidence’ Evaluation

We conducted a mixed‐methods study comprising a quantitative survey, direct observation of play performances, a qualitative survey and qualitative semi‐structured interviews. The evaluation was informed by the theoretical framework of acceptability [32] and principles of trauma‐informed co‐production [24].

#### 2.3.1 Quantitative Survey

Theatre company staff invited audience members to complete an anonymous paper questionnaire immediately following the play and collected completed forms (Appendix 1). The questionnaire was based on The Audience Experience Framework, which includes five dimensions: (a) engagement and concentration, (b) learning and challenge, (c) energy and tension, (d) shared experience and atmosphere and (e) personal resonance and emotional connection, with three questions per dimension, yielding a total of 15 questions. We used a 5‐point Likert scale to measure agreement with two opposing statements. The scores for the three questions for each dimension are summed, yielding a dimension score ranging from 5 to 15; the total score for the play is a sum of dimension scores [33] (Appendix 1). The questionnaire also included socio‐demographic questions (age, gender, ethnicity). The first author collected completed questionnaires from the theatre staff and entered responses into an Excel database.

#### 2.3.2 Direct Observation of Play Performances

The first and second authors observed one each out of four play performances and manually completed the semi‐structured observation schedule. The schedule was based on the five dimensions of The Audience Experience Framework [33]. Researchers met to discuss the schedule before conducting observations, to ensure reliability. The completed schedules were typed up and included in the qualitative data set.

#### 2.3.3 Qualitative Survey

The theatre company staff distributed their standard feedback form at the first two performances. For the second two performances, the standard feedback form was added to the end of the quantitative survey and distributed by researchers. Questions were open‐ended and explored audience motivations to attend the performance, emotional response, successful show elements, the value of community theatre, recommendations for next steps and any other feedback (Appendix 1). The first author received scanned completed feedback forms from the theatre staff and added them to the qualitative data set.

#### 2.3.4 Qualitative Semi‐Structured Interviews

The first author recruited a purposive sample of interview participants from three sources to capture experiences of the play team and audiences: questionnaire responders who agreed to be contacted about an interview, emailing the ‘Hard Evidence’ team and asking interviewees to share the information about the study (snowball sampling). The researcher emailed those who expressed interest, provided study details and arranged online interviews on Microsoft Teams with those willing to proceed. Interview topic guides were based on the seven component constructs of the theoretical framework of acceptability for healthcare interventions: affective attitude, burden, perceived effectiveness, ethicality, intervention coherence, opportunity costs and self‐efficacy [32]. Interviews were audio‐recorded, professionally transcribed, checked, anonymized and added to the qualitative data set.

#### 2.3.5 Analysis

We collected and analyzed quantitative and qualitative data separately and integrated findings using the ‘following a thread’ technique (Morran‐Ellis 2006). The first author analyzed quantitative survey responses in Excel with descriptive statistics (Appendix 2). The first and last authors analyzed all direct observation notes, qualitative survey responses and interview transcripts in one qualitative data set using the framework method [34]. The first author read and reread all qualitative data and the last author familiarized herself with 30% of the data set. Both researchers independently manually coded one set of observation notes, 16 survey responses and 4 transcripts in Excel. We used a combination of deductive and inductive coding. First, we coded all the data against the seven constructs of the theoretical framework of acceptability [32]. Then, we inductively coded data that did not map on the constructs. The researchers met to discuss and agree the initial coding framework, which the first author then applied to the rest of the data set. The framework was subjected to two iterations throughout the coding process. The first author developed and wrote descriptive accounts of the qualitative candidate themes.

Finally, the first author integrated quantitative results and qualitative candidate themes with the ‘following a thread’ technique [35, 36]. Integration according to this approach requires that the different methods are given equal weight in analysis, directed at the same research question or objective and are mutually dependent whilst keeping the integrity and modality of each [36]. ‘Following a thread’ consists of analyzing each data source from within its own modality to generate themes relevant to answering the research aims. Then, a theme from one data source is followed across to the others to create ‘a constellation of findings which can be used to generate a multi‐faceted picture of the phenomenon’ [36, p. 54]. The qualitative candidate themes that were much larger and richer became the candidate integrated themes, which we followed into the quantitative findings. We discussed the candidate integrated themes as a team. Our analytical thinking was informed by guidance for a trauma‐informed approach [24]. We developed final threads' themes that answered our research questions. The first author wrote descriptive accounts of the final analytical integrated themes (i.e., integrated threads).

### 2.4 Ethics Approval

The study received favourable opinion from the University of Bristol Faculty of Health Sciences Research Ethics Committee (30 June 2022, Ref: 11704). A proportionate approach to consent was granted for the audience surveys, and full verbal informed consent was obtained for qualitative interviews. Approval was also obtained to analyze qualitative survey data collected by the theatre company from the two pre‐study performances. Because of the topic of domestic abuse, we took measures to prevent re‐traumatization during qualitative interviews. Topic guides focused solely on the play, and the interviewer followed a distress protocol and signposted support services and resources to ensure that the individual's health and well‐being was protected.

## 3 Results

### 3.1 Co‐Development of Research Project Phase

In July 2022, nine individuals attended the participatory workshop: three researchers, one university communications specialist and members of the ‘Hard Evidence’ team – a theatre producer and three actors (two members of the coMforT study PPI group and one who was already working with the theatre company).

Based on the themes developed from the analysis of the workshop artefacts and fieldnotes, we made the following changes to the study protocol:

- Research aims to include the evaluating the safety of co‐developing and performing ‘Hard Evidence’.
- Topic guides to explore the impact and process of community theatre specifically.
- To understand the impact of the play in disseminating messages about PPI in research.
- To focus on the emotional dimension of audience responses to the play.

### 3.2 ‘Hard Evidence’ Evaluation

Between November 2021 and July 2022, we collected 20 responses to the quantitative survey and 56 responses to the qualitative survey, carried out 4.25 h of direct observations over two performances and interviewed 15 participants (7 audience members and 8 ‘Hard Evidence’ team members).

Of 15 interview participants, 13 were female; 14 were White and 1 was mixed/multiple ethnic groups. Two audience members interviewed disclosed experiences of domestic abuse, and another two disclosed supporting someone who had experienced domestic abuse. The mean age of the interview participants was 47 years (SD = 14.48, range 22 to 83).

Of 20 quantitative questionnaire responders, 16 were female; 18 were White, 1 was Asian and 1 ‘other ethnicity’. The mean age of the questionnaire responders was 48 years (SD = 18.01, range 22–83).

We developed three analytical themes with ten sub‐themes summarizing integrated qualitative and quantitative findings answering our research questions (Table 1).

**Table 1:** Themes supported by quantitative and qualitative data.

Integrated themes	Sub‐themes	Audience quantitative survey (*n* = 20)	Audience qualitative survey (*n* = 56)	Play observations (*n* = 2)	Semi‐structured interviews (*n* = 15)
1. Value of the play	1.1. What messages was the play successful in disseminating?	20 (Learning and Challenge dimension)	26	Obs1 Obs2	Audience members 1, 3, 5, 4, 5, 6 and 7, University Communications Specialist, Theatre Communications Specialist, Theatre Director, Researchers 1 and 2
	1.2. Who was the play successful in disseminating to?	20 (Personal Resonance and Emotional Connection dimension)		Obs1	Audience members 1, 3, 4, 5, 6 and 7, University Communications Specialist, Theatre Communications Specialist, Researchers 1 and 2, Actor 3
	1.3. How did play work in disseminating key messages?	20 (Engagement and Concentration dimension)	49	Obs1 Obs2	Audience members 2, 4 and 7. University Communications Specialist, Theatre Communications Specialist, Theatre Director, Researchers 1 and 2, Actors 1, 2 and 3
		20 (Energy and Tension dimension)			
2. The risks of re‐traumatization	2.1. The risk of re‐traumatizing the actors				Audience members 1, 3, 6 and 7. University Communications Specialist, Theatre Director, Researcher 1, Actors 1, 2 and 3
	2.2. The risk of re‐traumatizing the audiences		16	Obs1 Obs2	Audience members 1, 3, 4, 5 and 6. University Communications Specialist, Theatre Director, Researchers 1 and 2, Actors 2 and 3
3. Reducing the risks of re‐traumatization	3.1. Safety	20 (Shared Experience and Atmosphere dimension)		Obs1 Obs2	Audience members 2, 4, 5 and 6. University Communications Specialist, Theatre Director, Researchers 1 and 2, Actors 1, 2 and 3
	3.3. Trustworthiness and transparency, peer support and collaboration		9	Obs1 Obs2	Audience members 1, 4, 5, 6 and 7, University Communications Specialist, Theatre Communications Specialist, Theatre Director, Researchers 1 and 2, Actor 1
	3.4. Choice and empowerment		29		Audience members 3, 4, 5 and 6, University Communications Specialist, Theatre Communications Specialist, Theatre Director, Researchers 1 and 2, Actors 1 and 2
	3.5. Cultural consideration		44		Audience members 4, 5 and 6, University Communications Specialist, Theatre Director, Researcher 2, Actors 2 and 3

*Note:* The quantitative surveys column reports the number of responses for each dimension of the Audience Experience Framework by theme (Appendix 1). The qualitative surveys column reports the number of qualitative surveys responses that contributed to each theme. The observations and semi‐structured interviews columns report the observations and individual transcripts that contributed to each theme, respectively.

Abbreviation: *n*, number of survey responses.

#### 3.2.1 Value of the Play for Disseminating Research on a Sensitive Topic

Analysis indicated that the play had limited impact on the public's understanding of the value of PPI in research. However, it was effective in raising awareness about domestic abuse, because of how audiences emotionally engaged with the play.

##### 3.2.1.1 What Messages Was the Play Successful in Disseminating?

There were mixed views on the effectiveness of the play in raising awareness about PPI in research on domestic abuse. Only three questionnaires mentioned PPI, research or empowerment, and one audience member interview participant reported being unconvinced by the character of the researcher. However, some audience members were more positive about the effectiveness of the play in communicating messages about PPI – citing how informative it had been regarding PPI and its empowering impact on public contributors. The project team also had divided views on the plays' perceived impact in disseminating positive messaging around the value of PPI. Some team members thought that the play's message about the value of PPI was secondary to the message about the nature and impact of domestic abuse and sources of support. Others felt that it had been effective in showing the benefits of PPI – particularly in how it communicated these to an audience that might not typically engage in research.

I think maybe people who aren't involved in academia it can seem a bit like, ‘Oh, no, I couldn't get involved in a research project, not me’. Whereas I think it made it seem very accessible and actually showed the advantages of getting involved in research projects aren't just for the researcher. Audience Member 6

Both audience members and the project team saw the play as being effective in communicating messages about domestic abuse. Interviewees discussed its positive impact in raising awareness, challenging stereotypes through highlighting the diversity of experiences, emphasizing the power of support and expressing positive messages of strength, resilience and hope in recovery from abuse.

I think it was very successful in this way. It showed how strong these women are. Again, this friendship, and the desire and willingness to support and help others. And because they have their own lived experience, they know how to support without pushing, without pressing, without controlling. Just being there, always ready. Researcher 1

The Learning and Challenge dimension of the quantitative survey was rated highly by audience members (*M* = 12.9, SD = 2.17), with audiences answering that their eyes were opened to some new ideas (*M* = 4.6, SD = 0.68), felt provoked and challenged (*M* = 4.05, SD = 1.05) and that it got them thinking about things differently (*M* = 4.25, SD = 0.97) (Appendix 2). Qualitative survey respondents also focused on the play's success in discussing abuse, including raising awareness (*n* = 8), the importance of support and friendship (*n* = 7) and communicating hopeful, optimistic and empowering messages around abuse (*n* = 7). Observations of the Q&A sessions corroborated this, with comments including how informative the play had been, and the potential impact on encouraging people to get in touch with helplines.

##### 3.2.1.2 Who Was the Play Successful in Disseminating To?

Given that interviewees felt that the play was most successful in disseminating messages around recovery from abuse, it is unsurprising that they also saw it as vital that the play engaged with audience members who were abuse survivors. This meant that the depth or quality of engagement was seen as more important than the number of people it reached.

The smallest performance of the tour was regarded as having the biggest impact by people interviewed, because the actors were able to connect and engage with the audience, making it more intimate, intense and affecting. Audience members who disclosed that they were abuse survivors said that the play had been an incredibly powerful and emotional experience for them, feeling high levels of empathy for both the characters and actors and pride at their bravery, as well as connected to other audience members.

I found it incredibly moving, even when I got there, or at the end, just with the… Even those, like I say, eight or ten of us, just with everyone there, knowing that everyone had survived violence, I found it incredibly moving. I'd forgotten that feeling. Because it's so rare that you're in it, you're usually the only one, or you've got to keep it to yourself or whatever. We didn't talk about it, it was just knowing, just being around other people who were […] I found that really moving […] very strengthening. Audience Member 4

The play was also felt by all the project team to have had a positive impact on the two PPI actors, who both felt a sense of pride and achievement at having created something so impactful. One of the actors described how creating the play had given her space to address and resolve her trauma by re‐enacting it within a safe space.

Observations across both performances captured the diversity of audience experience, with three people crying in the performance, which interviewees felt had a large proportion of survivors, as well as the actors being visibly upset. Audience members tended to nod in agreement at specific lines, as well as discussing their own experiences of abuse with others following the play.

Given play audiences' heterogeneous experiences, the Personal Resonance and Emotional Connection survey dimension had a comparatively low total score and greater variation between individuals (*M* = 12.9, SD = 2.13). Audience members felt that they could really identify with the characters/story (*M* = 4.55, SD = 0.60) and found some aspects of the performance very moving (*M* = 4.7, SD = 0.47) but fewer audience members rated some aspects of the performance as seeming relevant to their own life (*M* = 3.65, SD = 1.34), the lowest score out of any question on the quantitative survey (Appendix 2).

##### 3.2.1.3 How Did the Play Work in Disseminating Key Messages?

In terms of its impact in disseminating messages around domestic abuse, the ‘Hard Evidence’ play was felt to be more powerful than reading scientific papers, as audiences were better able to emotionally connect to and identify with it, as well as being more accessible to those who might not typically engage with research.

Because there is that human connection of seeing something, and then feeling what is being portrayed, as opposed to just reading X amount of people in the UK were victims of domestic abuse, type thing. Audience Member 1

The audience members reported an unexpected strength of emotional response to the play, describing it as a powerful and often overwhelming experience. This was seen as being because (and not despite) of the fact that it was not ‘slick’; due to their lived‐experience, the actors were seen as being able to express ‘raw emotion’, and that audiences could empathize with their bravery, as well as the characters that they were playing. The project team were surprised at how many people cried at the show, but some felt that the play could have been more affecting because it was not sensationalist, melodramatic or overdone.

And the experience then, it was much more raw, I suppose, to have it as a community theatre. And to see those little forgetting of lines and stuff makes it much more‐ They're human, and it makes it much more immediate. And you empathise and sympathise with them much more as people, because you're seeing them there being all exposed and vulnerable, and doing this amazing thing. University Communications Specialist

This was echoed in the qualitative survey data; a large proportion of responders found it ‘emotional’ and felt ‘moved’ by the play, feeling that this stemmed from its authenticity (*n* = 17), how it involved real stories, emotions and issues (*n* = 12), enhanced by the intimate setting of the plays. Play observations noted high level of engagement: the lack of movement from audiences, who tended to lean forwards on their chairs or sat on the edge of their seat, some with their head either in their hands or gripping their chins.

Analysis of quantitative survey responses also found that the audiences had strong emotional engagement with the play. The mean value for the ‘Engagement and Concentration’ dimension of the Audience Experience Framework was 13.45 out of a possible 15 (SD = 2.01), with audiences rating that they felt completely absorbed by what was happening (*M* = 4.6, SD = 0.75), hardly noticed time passing (*M* = 4.45, SD = 1.13) and were often on the edge of their seat (*M* = 4.4, SD = 0.50). This was also consistent across the Energy and Tension dimension reported by audiences (*M* = 12.9, SD = 1.71), with audiences in general being gripped by the sights and sounds of the performance (*M* = 4.4, SD = 0.68), feeling lively and enthusiastic (*M* = 4.25, SD = 0.79) as well as tense and excited (*M* = 4.25, SD = 0.79) (Appendix 2).

#### 3.2.2 The Risks of Re‐Traumatization

The project team discussed a potential risk of re‐traumatizing the PPI actors through the process of creating and performing the play, as well as a lesser risk of distressing or re‐traumatizing audiences.

##### 3.2.2.1 The Risk of Re‐Traumatizing the Actors

The actors, wider project team and a family member supporting one of the actors described a range of negative emotional and health impacts experienced by the actors during the process. These occurred in response to the inherent risk of triggering the actors' traumatic histories through creating and performing the play, which, for one of them, led to re‐experiencing symptoms of pre‐existing post‐traumatic stress disorder. This mental health impact was exacerbated by the time burdens placed on the actors by requiring them to give up protective coping activities such as time with their friends, family and communities. There was also a secondary emotional burden placed on other team members who were worried about re‐traumatizing the actors. However, although this process was incredibly challenging, both actors felt that utilizing traumatic experiences to develop the play had been a valuable part of their healing process.

I remember the first night at [theatre base] and [actor] saying, in the Q&A, about muscle memory and that she relived it every time she performed ‘Hard Evidence’. I was horrified, I was utterly horrified. Researcher 2

So that kind of added the creativity, in terms of turning something that can feel quite negative inside your body into something that was actually positive. […] So I don't see that there was a negative side to it. It sounds like there was, and yes, I did go through a lot in terms of having my buttons pushed and having to deal with it. But at the same time, I've grown so much from it. Actor 1

##### 3.2.2.2 The Risk of Re‐Traumatizing the Audiences

Both the project team and audience members spoke about the possibility of emotional and mental health harms to play audiences. Many of the audience interviewed reported feeling sad, disheartened, worried and disappointed in response to what the characters in the play were going through, often crying during performances. Feeling sad, saddened or tearful was also common in the qualitative survey responses (*n* = 12) as well as feeling angry (*n* = 2), worried or anxious (*n* = 2), alone (*n* = 1), shocked (*n* = 1) and tired (*n* = 1). However, audience members, in general, thought that the play had been handled sensitively and avoided re‐traumatizing survivors of abuse. No participants reported feeling re‐traumatized or triggered by the play, despite many audience members interviewed self‐identifying as abuse survivors.

Yes, a lot of people cried… [p]eople would cry in the audience, so it shows, it was really interesting that people who had seen it before were still really affected. Actor 3

Getting the info across without being too traumatic, the delicate way it was handled. Questionnaire responder

#### 3.2.3 Reducing the Risks of Re‐Traumatization Through Applying Principles of Trauma‐Informed Co‐Production

Interview participants reported a range of strategies that the project team had either used or could have used that were aimed at reducing the risks of re‐traumatization through enhancing safety, trustworthiness and transparency, peer support, collaboration and mutuality, choice and empowerment and cultural considerations.

##### 3.2.3.1 Safety

Despite the difficult and negative emotions that the play elicited in some audience members, they described how feeling ‘held’ in a safe and containing space with other people reduced their fears about being triggered by the play. Because it was a live performance, audiences could share that experience with others (creating a sense of safety) as well as there being rituals around theatre‐going, which provided structure and emotional containment for a potentially distressing experience.

I think it's really important for the audience, especially if some of them are survivors of trauma or worked with trauma or just traumatised. It's really important for the theatre of it to make them safe so that you feel safe as an audience member. […] People who are good at putting on theatre do that anyway; they make everyone feel safe […] The doors are closed. The lights go down, you're safe, and nothing is going to interrupt your thing. Audience Member 4

Play was seen as a key part of creating safety for the actors in rehearsals, which were described as characterized by fun, laughter, joking, warmth and loveliness, something the director said they had deliberately cultivated. Joking in rehearsals seemed to be a key coping mechanism for managing any difficult or triggering memories that had been brought up by ‘Hard Evidence’ for the two actors especially, who described it as something that could ‘override’ trauma responses. Laughter and joking were also key in the performance of the play; observations noted how audience tension was diffused by laughter following a joke on stage.

[W]e'd just start giggling. We felt disrespectful at times, because of the subject, but it was our way of making it easier for ourselves […] So, our way of doing it, because there were only two of us and we got really close, was just to giggle about things […] I think that was our way of coping. Actor 2

The importance of people sharing an experience in this way was something corroborated by quantitative survey responses, with people rating the Shared experience and Atmosphere dimension of the survey highly (*M* = 13.55, SD = 1.36). Audiences answered positively about noticing a real buzz in the audience (*M* = 4.3, SD = 0.73), rating particularly highly that it felt good to be sharing the experience with other people (*M* = 4.65, SD = 0.59), and that they would be talking about the experience for some time to come (*M* = 4.6, SD = 0.68) (Appendix 2). Observations from the smaller performance describe how people who did not know each other started talking about the play, as well as conversations (and hugs) between audience members and actors. In the larger performance, observations were noted of a ‘buzz’ afterward, with lots of conversations, and some audience members comforting each other.

##### 3.2.3.2 Trustworthiness and Transparency, Peer Support and Collaboration

The most important facilitator of a sense of self‐efficacy for project team members as well as audiences was the trusting relationships with others. This included the emotionally supportive friendship that developed between the two PPI actors, as well as other project team members' high levels of trust in the theatre company, which enabled them to take risks on the project. Audience members' familiarity with the theatre company also increased their confidence, as they both knew the space, and trusted the theatre company not to produce a play that might be traumatizing for them.

Several members of the project team said that they would have valued working alongside a domestic abuse organization on this project. Their role would be to provide specialist supervision and mentoring to the project team, as well as direct support to audience members at performances, including taking referrals.

Yes. Thankfully, touch wood, they developed a really supportive group between them, and they were really strong women, as well, who were at a good place in their journeys. [B]ecause it could have gone horribly wrong really, on reflection. Researcher 2

So, if there were things that came up in sessions, bits of stories which did come up of the women's experience, that you obviously didn't put in the play but things that [it would just trigger] and we would talk about or‐ So just that kind of mentoring. Having someone to just speak those things that were coming up. Theatre Director

Key to fostering trusting collaborative relationships was funding, as noted by most of the project team members, who emphasized the importance of funding (particularly travel and salaries), to ensure that those with lived experience of abuse were properly supported in working on a potentially re‐traumatizing project.

[T]he main lesson which I learned from this experience is that proper funding, staff, and resourcing is the key. It is important, first of all, to support people with lived experience, that's no question. But also, to support everyone who supports them and who supports the process, in a way that it is equal, fair, and just to everyone. Researcher 1

##### 3.2.3.3 Choice and Empowerment

All members of the project team and audience members interviewed saw the ‘Hard Evidence’ play as being strongly aligned with their values – specifically the focus on supporting survivors' autonomy, choice and empowerment and its hopeful, strengths‐based message. The project team also felt that the process creating the play had been in line with their ethical views: preserving the actors' autonomy in how they told their stories, making sure that the actors were supported and ensuring that the play had a positive impact on the project team.

[The play] is giving a voice to people who seem voiceless, and I think that that is a really important thing. And I find that really motivating, I suppose. So yes, it fits really well with my values. University Communications Specialist

Relatedly, audience interviewees said that they felt empowered by watching the play, that it made them feel hopeful and optimistic that people in difficult situations could get out with the right support, with survey responders also feeling hopeful, empowered as well as uplifted and heartened by the play.

I just felt really proud of them […] And I think even got tearful at the end. Precisely because they were not professional actors, they just gave themselves. And they were struggling with the acting, at some point. I think it was really beautiful, and really empowering for them. And really, fantastically moving to watch. Audience Member 3

Both PPI actors also found the experience of being part of ‘Hard Evidence’ personally empowering, despite initial crises of confidence around performing. Both the actors felt that their self‐efficacy had increased during the project, positively affecting their confidence and self‐worth in other areas of life.

Part of the process of what I do understand and what I have learnt, and reflecting back, is that my self‐esteem, my self‐confidence, and my self‐worth have definitely developed. And my personality has become stronger, because of going through that process, as well. Actor 1

##### 3.2.3.4 Cultural Consideration: Creating an Accessible Play

The fact that ‘Hard Evidence’ was a piece of community theatre was seen to have broadened its reach, increasing accessibility to audiences who would not typically go to the theatre. Accessibility was key for the play to be effective in building a sense of community, intimacy and closeness between audience members, and enabling survivors to connect to others. These benefits to community theatre were enhanced by having a Q&A after ‘Hard Evidence’ performances.

And I think the importance of it being community theatre, once again it's not just your average theatre goer who would come. It's those people in the community who really is who we're trying to engage. If you put a document through their door with all the PPI information would probably say like, ‘Absolutely not, I don't want to go’. Audience Member 06

[The main point of the community play was] so people don't feel alone or ashamed. Survey responder 103

## 4 Discussion

This mixed‐methods study evaluated the impact, acceptability and safety of a play co‐produced with PPI contributors in disseminating research about domestic abuse. Our analysis indicated that although audience members and the play team felt that it had been impactful at increasing audience knowledge and changing attitudes about domestic abuse, there was a mixed reception about its impact at communicating messages about PPI in research.

Co‐development and dissemination of a play with PPI contributors with lived experience carries a risk of harm. Actors can be re‐traumatized through reliving their experiences during the process of creating and performing the play. The public can be distressed and re‐triggered through engaging with the play and reminders about their own traumatic experiences. The play creators tried to prevent and mitigate the risk of harm through creating safe environments, centring trusting relationships, providing peer support and opportunities for choice and empowerment and making the play accessible.

The impact of ‘Hard Evidence’ in successfully disseminating messages around domestic abuse was seen to be due to its enhanced ability to emotionally engage the public over traditional dissemination methods. This ability to engage audiences in health research through their emotional responses to the play is something reported in other theatre‐based dissemination [15, 16, 37], possibly due to theatre's ability to ‘communicate research findings in an emotive and embodied manner’ [38]. What is unique in our findings is that audiences emotionally engaged not just with the vulnerability of the characters but also the PPI actors, knowing that the play was based on personal experiences, resulting in increased audience feelings of compassion towards the actors. Although the ‘Hard Evidence’ play was not directly based on specific experiences of domestic abuse, the emotional process performed by the PPI actors can be understood as a form of emotional labour involving ‘deep acting’ [39, p. 38]. Performing the play often led them to access genuine and often difficult emotions, as opposed to simulating them. Using theatre to re‐enact traumatic memories as a way of healing from them is a technique common within trauma‐informed drama therapy [40, 41, 42, 43], and there is an evidence base around the effectiveness of this for survivors of domestic abuse specifically [44, 45]. Importantly, however, the development of the play was not facilitated by a therapist, and two members of the project team interviewed stated that they would have felt more comfortable in their roles if they had supervision from a therapist experienced in this area, appropriate supervision being a key part of trauma‐informed ‘systems of care’ [46].

Our finding on the limited impact of the play on raising audience awareness about PPI in research can be explained by several reasons. Frameworks, guidance and tools designed to support researchers in planning, delivering and evaluation health research dissemination frequently emphasize the importance of determining target‐audiences before engaging in dissemination [47, 48]. However, this is problematized when dissemination is co‐produced with public contributors who might have different aims for their output than funders or health researchers [6]. Although the project funder wanted to prioritize messaging around the value of PPI in the co‐produced output, the play development was led by the PPI contributors with lived experience who prioritized the storyline about domestic abuse, resilience and peer support. Part of the issue could also have been that the coMforT study researcher was not involved in the co‐production process at the same level as PPI contributors with lived experience.

We found that the way the theatre company worked with the actors to prevent harm adhered to principles of trauma‐informed co‐production and dissemination [24]. This included things like creating rituals around the theatre to enshrine it as a safe space [43, 49, 50, p. 365], introducing an element of play into their work, basing relationships on trust, collaboration and empowerment [6, 24] and working directly with communities in a culturally appropriate way [24, 51, 52]. However, several of our project team interviewees also commented on the value of input by the researchers in ensuring that the play was empowering, hopeful, centred on the women's strength and was not going to be sensationalist or triggering to audience members who had experienced domestic abuse.

### 4.1 Strengths and Limitations

A key strength of this study was the PPI work undertaken in refining the research aims and developing the protocol – involving the whole ‘Hard Evidence’ project team. This was facilitated by having a researcher with PPI expertise in the research team. This work resulted in a focus on how the potential risk of re‐traumatizing team members and audiences could be prevented and mitigated.

This study drew on several theoretical frameworks in understanding the impact and acceptability of a play as a format for dissemination, including the Audience Experience Framework for audience experience [33], the theoretical framework of acceptability [32] and the six principles of trauma‐informed co‐production [24]. The framework method [34] and ‘following a thread’ approach enabled the team to work collaboratively to develop and test the coding framework, ensuring that the integrated analytical themes were underpinned by the data.

The main limitation of this study caused by the funding constraint is the small sample size, for both the qualitative interviews and the quantitative and qualitative surveys, as well as observing only two out of four play performances. This was partly because the research project started after the first two performances of the play, which had larger audiences. We evaluated these first two performances using the theatre company's own qualitative survey. We used the study quantitative survey for the later performances, which had smaller audiences. As a result, the quantitative survey sample of 20 was much smaller than the qualitative survey sample of 56. Despite optimizing recruitment of interview participants through snowballing and offering survey respondents an opportunity to opt in, we could recruit only 15 individuals within the study time frame.

### 4.2 Implications for Future Research and Practice

This study is specific to the experience of co‐developing a play with PPI contributors as a way of disseminating research on domestic abuse. Although it seems plausible that many of the findings would also apply to plays created about other sensitive subjects – this would need to be evaluated. It would also be valuable to understand how the impact and potential risks of a play compared to other performance media as a format for dissemination such as dance, live comedy and stand‐up poetry.

The key learning points for practitioners in this area are that adequate resources should be allocated, and care should be taken in using plays for dissemination of research on sensitive subjects to prevent re‐traumatizing audiences and project team members, particularly those with lived experience of the sensitive subject, to protect their health and well‐being. Organizations who have specialist expertise in supporting individuals should be sought as collaborators, to provide ongoing psychological support to the project team, as well as attending performances and providing drop‐in support, signposting and helping create a quiet, psychologically safe space for audience members who are distressed. Adequate resources are vital to ensuring that everyone can be supported in their role within the project. Practitioners should also ensure that the creative process centres play, empowerment and safety and the play is not based on details of individuals' personal experiences.

## 5 Conclusions

Co‐developing plays with PPI contributors with lived experience can be an acceptable and safe format for disseminating research on sensitive subjects if the following resources and conditions are in place. The team should be given flexibility in determining the key messages that they want to disseminate, giving PPI contributors autonomy in this process. The funder should be made aware that the output will be shaped by the PPI contributors. The co‐development process should centre around trauma‐informed co‐production principles to prevent re‐traumatizing both audience members and the project team, including ensuring psychological safety in rehearsals and performances, providing resources to enable the development of supportive and trusting relationships within the project team, centering PPI contributors' choices and creating a play that is accessible to community members.

## Author Contributions

**Cat Papastavrou Brooks:** conceptualization, investigation, methodology, writing–review and editing, writing–original draft, data curation, formal analysis. **Noreen Hopewell‐Kelly:** conceptualization, funding acquisition, writing–review and editing, methodology, supervision, investigation. **Natalia V. Lewis:** conceptualization, investigation, funding acquisition, writing–original draft, writing–review and editing, data curation, supervision, methodology.

## Ethics Statement

The study received favourable opinion from the University of Bristol Faculty of Health Sciences Research Ethics Committee (30 June 2022, Ref: 11704). A proportionate approach to consent was granted for the audience surveys, and full verbal informed consent was obtained for qualitative interviews. Approval was also obtained to analyze qualitative survey data collected by the theatre company from the two pre‐study performances. Because of the topic of domestic abuse, we took measures to prevent re‐traumatization during qualitative interviews. Topic guides focused solely on the play; the interviewer followed a distress protocol and signposted support services and resources.

## Conflicts of Interest

The authors declare no conflicts of interest.

## Data Availability

Due to the sensitivity of the data involved, these data are published as a controlled data set at the University of Bristol Research Data Repository data.bris, at https://doi.org/10.5523/bris.3vj9nmc9bekm62pvlyi452jlv2. The metadata record published openly by the repository at this location clearly states how data can be accessed by bona fide researchers. Requests for access will be considered by the University of Bristol Data Access Committee, who will assess the motives of potential data re‐users before deciding to grant access to the data. No authentic request for access will be refused and re‐users will not be charged for any part of this process [53].

## Appendix 1 Audience Quantitative Survey

### 1.1 Hard Evidence Audience Survey

The Hard Evidence play was developed by women with lived experience of domestic abuse who had been involved in a research project (coMforT), researchers, actors and a theatre producer. We want to understand more about what was good about the way in which this play was developed and the impact that it might have had on people watching it. We also want to develop some guidance for people who might want to work in a similarly collaborative way to the Hard Evidence team.

As part of this process, we are asking audience members at Hard Evidence to answer some questions about their experience of the play.

Your responses will be kept confidential, and only the research team will have access to your data. Anonymous quotes may be published, but no names or identifying details will be reported, so it will not be possible to trace who said them. We are also interested in talking to people who would be prepared to discuss their experiences of the play further. If that is something you would also be interested in or want to hear about the findings of this research, please fill in your details below.

If you have any concerns about any part of the study, please contact the lead researcher, Natalia Lewis, on Nat.Lewis@bristol.ac.uk. If you wish to make a complaint, please contact research-governance@bristol.ac.uk.

‐‐‐‐‐‐‐‐‐‐‐‐‐‐‐‐‐‐‐‐‐‐‐‐‐‐‐‐‐‐‐‐‐‐‐‐‐‐‐‐‐‐‐‐‐‐‐‐‐‐‐‐‐‐‐‐‐‐‐‐‐‐‐‐‐‐‐‐‐‐‐‐‐‐‐‐‐‐‐‐‐‐‐‐‐‐‐‐‐‐‐‐‐‐‐‐

Please circle the number that best fits with your experience watching the play:

**Table:**

**My concentration was wandering**	1□	2□	3□	4□	5□	**I was completely absorbed by what was happening**
**It felt like time was passing slowly**	1□	2□	3□	4□	5□	**I hardly noticed the time passing**
**The performance did not really hold my attention**	1□	2□	3□	4□	5□	**I was often on the edge of my seat**
**I did not feel like I was learning anything**	1□	2□	3□	4□	5□	**My eyes were opened to some new ideas**
**I was mostly in my ‘comfort zone’**	1□	2□	3□	4□	5□	**I felt challenged and provoked**
**There was nothing much new for me**	1□	2□	3□	4□	5□	**It got me thinking about things differently**
**It did not really get me going**	1□	2□	3□	4□	5□	**I was gripped by the sights and sounds of the performance**
**I felt tired and uninterested**	1□	2□	3□	4□	5□	**I felt lively and enthusiastic**
**I felt flat**	1□	2□	3□	4□	5□	**I felt tense and excited**
**There was not much sense of atmosphere**	1□	2□	3□	4□	5□	**I noticed a real buzz in the audience**
**I did not feel much connection with other audience members**	1□	2□	3□	4□	5□	**It felt good to be sharing the experience with other people**
**I do not feel much urge to discuss the performance**	1□	2□	3□	4□	5□	**I will be talking about the experience for some time to come**
**I did not feel much connection with the characters/story**	1□	2□	3□	4□	5□	**I felt I could really identify with the characters/story**
**There was not really much that touched me**	1□	2□	3□	4□	5□	**I found some aspects of the performance very moving**
**It did not say much about my life or experiences**	1□	2□	3□	4□	5□	**Some aspects of the performance seemed relevant to my own life**

### 1.2 About You

Age (full years): ___________________________________

How would you describe your gender:

1. Female (including transgender women) □
2. Male (including transgender men) □
3. Prefer to self‐describe as ______________________ (non‐binary, gender‐fluid, agender, please specify)

What is your ethnic group:

1. White □
2. Mixed/multiple ethnic groups □
3. Asian/Asian British □
4. Black/African/Caribbean/Black British □
5. Other ethnic group □_____________________________________

### 1.3 Feedback About the Show

1. Why did you come to watch this show? (tick all that apply):
  a. Friend or family of cast □
  b. Professional/academic involvement □
  c. Regular ACTA show audience member □
  d. Interested in subject of surviving domestic abuse □
  e. Other (please state) __________________________________
2. How did the show make you feel? Please write three words! ________________________________________________
3. What were the most successful elements of this show for you, and did you learn anything? ______________________________________________________________________________________________________________________________________________
4. What are the benefits of hearing stories told by community performers? ________________________________________________________________________________________________________________________________________________________
5. Any thoughts on what we should do next, to develop this show or to create a new one? performers? ______________________________________________________________________________________________________________________________________
6. Any other feedback?__________________________________________________________________________________________________________________________________________________________

### 1.4 Talking About the Show

I am happy to be contacted for a follow‐up chat about the Hard Evidence Play □

I would like to be sent a report of the main findings of this study □

My contact details are (phone or email): ___________________________________________________________

## Appendix 2 Audience Experience quantitative survey results (*n* = 20)

**Table:**

Engagement and concentration	Mean*	Median*	SD
My concentration was wandering/I was completely absorbed by what was happening	4.6	5	0.75
It felt like time was passing slowly/I hardly noticed the time passing	4.45	5	1.15
The performance did not really hold my attention/I was often on the edge of my seat	4.4	4	0.50
*Total dimension score*	13.45	14	2.01
Learning and challenge			
I did not feel like I was learning anything/My eyes were opened to some new ideas	4.6	5	0.68
I was mostly in my ‘comfort zone’/I felt challenged and provoked	4.05	4	1.05
There was nothing much new for me/It got me thinking about things differently	4.25	5	0.97
*Total dimension score*	12.9	13.5	2.17
Energy and tension			
It did not really get me going/I was gripped by the sights and sounds of the performance	4.4	4.5	0.68
I felt tired and uninterested/I felt lively and enthusiastic	4.25	4	0.79
I felt flat/I felt tense and excited	4.25	4	0.79
*Total dimension score*	12.9	13	1.71
Shared experience and atmosphere			
There was not much sense of atmosphere/I noticed a real buzz in the audience	4.3	4	0.73
I did not feel much connection with other audience members/It felt good to be sharing the experience with other people	4.65	5	0.59
I do not feel much urge to discuss the performance/I will be talking about the experience for some time to come	4.6	5	0.68
*Total dimension score*	13.55	14	1.36
Personal resonance and emotional connection			
I did not feel much connection with the characters/story/I felt I could really identify with the characters/story	4.55	5	0.60
There was not really much that touched me/I found aspects of the performance very moving	4.7	5	0.47
It did not say much about my life or experiences/Some aspects of the performance seemed relevant to my own life	3.65	4	1.35
*Total dimension score*	12.9	13.5	2.13
*Total measure score***	118.5	120	11.30

*A higher mean/median on each dimension indicates success of the play along that dimension (between 1 and 5).

**The total score for the play is a sum of dimension scores, ranging from 25 to 75.

## References

[1] NIHR . How to Disseminate Your Research (2019), accessed November 4, 2024, https://www.nihr.ac.uk/documents/how-to-disseminate-your-research/19951.

[2] P. M. Wilson , M. Petticrew , M. W. Calnan , and I. Nazareth , “Does Dissemination Extend Beyond Publication: A Survey of a Cross Section of Public Funded Research in the UK,” Implementation Science 5, no. 1 (2010): 61, 10.1186/1748-5908-5-61. 20682083 PMC2922079

[3] J. Kerner , B. Rimer , and K. Emmons , “Introduction to the Special Section on Dissemination: Dissemination Research and Research Dissemination: How Can We Close the Gap?,” *Health Psychology* 24, no. 5 (2005): 443–446, 10.1037/0278-6133.24.5.443. 16162037

[4] T. L. Mayo‐Gamble , J. Cunningham‐Erves , C. Kas‐Osoka , G. W. Johnson , N. Frazier , and Y. Joosten , “A Multiperspective on the Broad Dissemination of Research Findings to Past Research Participants and the Community‐At‐Large,” Translational Behavioral Medicine 12, no. 1 (2022): ibab095, 10.1093/tbm/ibab095. 34244808

[5] S. Cyril , B. J. Smith , A. Possamai‐Inesedy , and A. M. N. Renzaho , “Exploring the Role of Community Engagement in Improving the Health of Disadvantaged Populations: A Systematic Review,” Global Health Action 8, no. 1 (2015): 29842, 10.3402/gha.v8.29842. 26689460 PMC4685976

[6] B. McDavitt , L. M. Bogart , M. G. Mutchler , et al., “Dissemination as Dialogue: Building Trust and Sharing Research Findings Through Community Engagement,” Preventing Chronic Disease 13 (2016): 150473, 10.5888/pcd13.150473. PMC4797478 26986541

[7] C. E. Knoepke , M. P. Ingle , D. D. Matlock , R. C. Brownson , and R. E. Glasgow , “Dissemination and Stakeholder Engagement Practices Among Dissemination & Implementation Scientists: Results From an Online Survey,” PLoS One 14, no. 11 (2019): e0216971, 10.1371/journal.pone.0216971. 31721784 PMC6853327

[8] D. M. McNeal , R. E. Glasgow , R. C. Brownson , et al., “Perspectives of Scientists on Disseminating Research Findings to Non‐Research Audiences,” Journal of Clinical and Translational Science 5, no. 1 (2021): e61, 10.1017/cts.2020.563. PMC8057369 33948281

[9] V. Minogue , M. Morrissey , and A. Terres , “Supporting Researchers in Knowledge Translation and Dissemination of Their Research to Increase Usability and Impact,” Quality of Life Research 31, no. 10 (2022): 2959–2968, 10.1007/s11136-022-03122-1. 35303224

[10] K. Boydell , B. M. Gladstone , T. Volpe , B. Allemang , and E. Stasiulis , “The Production and Dissemination of Knowledge: A Scoping Review of Arts‐Based Health Research,” Forum Qualitative Sozialforschung/Forum: Qualitative Social Research 13, no. 1 (2012): 32, 10.17169/fqs-.1.1711.

[11] K. D. Fraser and F. al Sayah , “Arts‐Based Methods in Health Research: A Systematic Review of the Literature,” Arts & Health 3, no. 2 (2011): 110–145, 10.1080/17533015.2011.561357.

[12] D. Lafrenière , S. Cox , G. Belliveau , and G. Lea , “Performing the Human Subject: Arts‐Based Knowledge Dissemination in Health Research,” Journal of Applied Arts & Health 3 (2013): 243–257, 10.1386/jaah.3.3.243_1.

[13] K. Rieger and A. S. H. Schultz , “Exploring Arts‐Based Knowledge Translation: Sharing Research Findings Through Performing the Patterns, Rehearsing the Results, Staging the Synthesis,” Worldviews on Evidence‐Based Nursing 11, no. 2 (2014): 133–139, 10.1111/wvn.12031. 24597543

[14] E. McNichol and P. Grimshaw , “An Innovative Toolkit: Increasing the Role and Value of Patient and Public Involvement in the Dissemination of Research Findings,” International Practice Development Journal 4, no. 1 (2014): 1–14, 10.19043/ipdj.41.008.

[15] R. Gray , M. Fitch , M. LaBrecque , and M. Greenberg , “Reactions of Health Professionals to a Research‐Based Theatre Production,” Journal of Cancer Education 18, no. 4 (2003): 223–229, 10.1207/s15430154jce1804_10. 14766333

[16] R. Gray , C. Sinding , V. Ivonoffski , M. Fitch , A. Hampson , and M. Greenberg , “The Use of Research‐Based Theatre in a Project Related to Metastatic Breast Cancer,” Health Expectations 3, no. 2 (2000): 137–144, 10.1046/j.1369-6513.2000.00071.x. 11281920 PMC5080956

[17] E. Asante , “Theatre: An Innovative Approach To Public Health Education,” *International Journal of Innovative Research and Advanced Studies* 3, no. 7 (2016): 28–34.

[18] L. A. Tarasoff , R. Epstein , D. C. Green , S. Anderson , and L. E. Ross , “Using Interactive Theatre to Help Fertility Providers Better Understand Sexual and Gender Minority Patients,” Medical Humanities 40, no. 2 (2014): 135–141, 10.1136/medhum-2014-010516. 25005177

[19] A. Colantonio , P. C. Kontos , J. E. Gilbert , K. Rossiter , J. Gray , and M. L. Keightley , “After the Crash: Research‐Based Theater for Knowledge Transfer,” Journal of Continuing Education in the Health Professions 28, no. 3 (2008): 180–185, 10.1002/chp.177. 18712795

[20] P. C. Kontos and G. Naglie , “Expressions of Personhood in Alzheimer's Disease: An Evaluation of Research‐Based Theatre as a Pedagogical Tool,” Qualitative Health Research 17, no. 6 (2007): 799–811, 10.1177/1049732307302838. 17582022

[21] M. Stuttaford , C. Bryanston , G. L. Hundt , M. Connor , M. Thorogood , and S. Tollman , “Use of Applied Theatre in Health Research Dissemination and Data Validation: A Pilot Study From South Africa,” Health: An Interdisciplinary Journal for the Social Study of Health, Illness and Medicine 10, no. 1 (2006): 31–45, 10.1177/1363459306058985. PMC2830105 16322042

[22] Substance Abuse and Mental Health Services Administration . *SAMHSA's Concept of Trauma and Guidance for a Trauma‐Informed Approach* (Rockville, MD: Substance Abuse and Mental Health Services Administration, 2014).

[23] S. Lonbay , A. Pearson , E. Hamilton , P. Higgins , E. Foulkes , and M. Glascott , “Trauma Informed Participatory Research: Reflections on Co‐Producing a Research Proposal,” Gateways: International Journal of Community Research and Engagement 14, no. 1 (2021): 1–8, 10.3316/informit.957734740832271.

[24] H. McGeown , L. Potter , T. Stone , et al., “Trauma Informed Co‐Production: Collaborating and Combining Expertise to Improve Access to Primary Care With Women With Complex Needs,” Health Expectations 26, no. 5: 1895–1914, 10.1111/hex.13795. PMC10485347 37430474

[25] C. Shimmin , K. D. M. Wittmeier , J. G. Lavoie , E. D. Wicklund , and K. M. Sibley , “Moving Towards a More Inclusive Patient and Public Involvement in Health Research Paradigm: The Incorporation of a Trauma‐Informed Intersectional Analysis,” BMC Health Services Research 17, no. 1 (2017): 539, 10.1186/s12913-017-2463-1. 28784138 PMC5547533

[26] T. H. DeRoy , “Narrative Justice Playmaking: A Toolkit for Transformative Theatre” (PhD Thesis, University of Kansas, 2023), https://search.proquest.com/openview/6d76b114f8a8bbedd53189543ae21cab/1?pq-origsite=gscholar&cbl=18750&diss=y&casa_token=1YDsmO4PTJ8AAAAA:CrWMvAoQslOfUZ6SpsROTNmamxAyW2O8nK4VorGyUy_wbfXtPAKruvVe9kC9C1EwO5WvegZ9BQ.

[27] M. Palidofsky and B. C. Stolbach , “Dramatic Healing: The Evolution of a Trauma‐Informed Musical Theatre Program for Incarcerated Girls,” Journal of Child & Adolescent Trauma 5, no. 3 (2012): 239–256, 10.1080/19361521.2012.697102.

[28] M. McKeown , G. Thomson , A. Scholes , et al., “Restraint Minimisation in Mental Health Care: Legitimate or Illegitimate Force? An Ethnographic Study,” Sociology of Health & Illness 42, no. 3 (2020): 449–464. 31657030 10.1111/1467-9566.13015

[29] N. V. Lewis , A. Gregory , G. S. Feder , et al., “Trauma‐Specific Mindfulness‐Based Cognitive Therapy for Women With Post‐Traumatic Stress Disorder and a History of Domestic Abuse Intervention Refinement and a Randomised Feasibility Trial (coMforT Study),” Pilot and Feasibility Studies 9 (2023): 112, https://orca.cardiff.ac.uk/id/eprint/160420/. 37400911 10.1186/s40814-023-01335-w PMC10316568

[30] M. Farr , P. Davies , H. Andrews , D. Bagnall , E. Brangan , and R. Davies , “Co‐Producing Knowledge in Health and Social Care Research: Reflections on the Challenges and Ways to Enable More Equal Relationships,” Humanities and Social Sciences Communications 8, no. 1 (2021): 105, 1, 10.1057/s41599-021-00782-1.

[31] A. Gibson , J. Welsman , and N. Britten , “Evaluating Patient and Public Involvement in Health Research: From Theoretical Model to Practical Workshop,” Health Expectations 20, no. 5 (2017): 826–835, 10.1111/hex.12486. 28664563 PMC5600246

[32] M. Sekhon , M. Cartwright , and J. J. Francis , “Acceptability of Healthcare Interventions: An Overview of Reviews and Development of a Theoretical Framework,” BMC Health Services Research 17, no. 1 (2017): 88, 10.1186/s12913-017-2031-8. 28126032 PMC5267473

[33] NEF , Capturing the Audience Experience: A Handbook for the Theatre (London: NEF, ITC, SOLT & TMA, 2008), accessed 2th November 2024, https://www.culturehive.co.uk/wp-content/uploads/2020/10/Capturing-the-audience-experience-A-handbook-for-the-theatre-1.pdf.

[34] N. K. Gale , G. Heath , E. Cameron , S. Rashid , and S. Redwood , “Using the Framework Method for the Analysis of Qualitative Data in Multi‐Disciplinary Health Research,” BMC Medical Research Methodology 13, no. 1 (2013): 117, 10.1186/1471-2288-13-117. 24047204 PMC3848812

[35] C. M. Dupin and G. Borglin , “Usability and Application of a Data Integration Technique (Following the Thread) for Multi‐ and Mixed Methods Research: A Systematic Review,” International Journal of Nursing Studies 108 (2020): 103608, 10.1016/j.ijnurstu.2020.103608. 32454297

[36] J. Moran‐Ellis , V. D. Alexander , A. Cronin , et al., “Triangulation and Integration: Processes, Claims and Implications,” Qualitative Research 6, no. 1 (2006): 45–59, 10.1177/1468794106058870.

[37] J. Lapum , P. Ruttonsha , K. Church , T. Yau , and A. M. David , “Employing the Arts in Research as an Analytical Tool and Dissemination Method: Interpreting Experience Through the Aesthetic,” Qualitative Inquiry 18, no. 1 (2012): 100–115, 10.1177/1077800411427852.

[38] K. Rossiter , P. Kontos , A. Colantonio , J. Gilbert , J. Gray , and M. Keightley , “Staging Data: Theatre as a Tool for Analysis and Knowledge Transfer in Health Research,” Social Science & Medicine (1982) 66, no. 1 (2008): 130–146, 10.1016/j.socscimed.2007.07.021. 17850943

[39] A. Hochschild , The Managed Heart: Commercialization of Human Feeling (3rd Edition) (Berkeley: University of California Press, 2012).

[40] S. de Smet , C. Rousseau , C. Stalpaert , and L. D. Haene , “A Qualitative Analysis of Coping With Trauma and Exile in Applied Theatre With Syrian Refugees: The Role of Within‐Group Interactions,” Arts in Psychotherapy 66 (2019): 101587, 10.1016/j.aip.2019.101587.

[41] Y. Kurahashi , “Theatre as the Healing Space: Ping Chong's Children of War,” Studies in Theatre and Performance 24, no. 1 (2004): 23–36, 10.1386/stap.24.1.23/0.

[42] M. L. Mondolfi Miguel and M. Pino‐Juste , “Therapeutic Achievements of a Program Based on Drama Therapy, the Theater of the Oppressed, and Psychodrama With Women Victims of Intimate Partner Violence,” Violence Against Women 27, no. 9 (2021): 1273–1296, 10.1177/1077801220920381. 32573368

[43] N. Sajnani and D. R. Johnson , Trauma‐Informed Drama Therapy: Transforming Clinics, Classrooms, and Communities (Springfield, Illinois: Charles C Thomas Publisher, 2014).

[44] H. Barnes and D. Peters , “Translating Trauma: Using Arts Therapies With Survivors of Violence,” South African Theatre Journal 16, no. 1 (2002): 157–183, 10.1080/10137548.2002.9687747.

[45] J. Campbell Kirk , “Dramatherapy With Women Survivors of Domestic Abuse: A Small Scale Research Study,” Dramatherapy 37, no. 1 (2015): 28–43, 10.1080/02630672.2015.1101480.

[46] R. Berger and L. Quiros , “Supervision for Trauma‐Informed Practice,” Traumatology 20 (2014): 296–301, 10.1037/h0099835.

[47] S. Kuruvilla , N. Mays , A. Pleasant , and G. Walt , “Describing the Impact of Health Research: A Research Impact Framework,” BMC Health Services Research 6, no. 1 (2006): 134, 10.1186/1472-6963-6-134. 17049092 PMC1635046

[48] J. Lavis , S. Ross , C. McLeod , and A. Gildiner , “Measuring the Impact of Health Research,” Journal of Health Services Research & Policy 8, no. 3 (2003): 165–170, 10.1258/135581903322029520. 12869343

[49] M. Redfern , “Safe Spaces and Scary Encounters: Core Therapeutic Elements of Trauma‐Informed Dramatherapy,” in Trauma Informed Drama Therapy: Transforming Clinics, Classrooms, and Communities (2014), 365–388.

[50] M. J. Richardson , “Theatre as Safe Space? Performing Intergenerational Narratives With Men of Irish Descent,” Social & Cultural Geography 16, no. 6 (2015): 615–633, 10.1080/14649365.2014.998269.

[51] S. C. Bodison , I. Sankaré , H. Anaya , et al., “Engaging the Community in the Dissemination, Implementation, and Improvement of Health‐Related Research,” Clinical and Translational Science 8, no. 6 (2015): 814–819, 10.1111/cts.12342. 26546337 PMC4819993

[52] S. Sawyer , S. M. Grieb , A. Long , et al., “Improving Research Dissemination to Black Sexual Minority Men: Development of a Community‐Led and Theory‐Based Dissemination Plan,” Health Promotion Practice 24, no. 1 (2023): 144–152, 10.1177/15248399211048462. 34628974

[53] N. Lewis and C. Papastavrou Brooks , Hard Evidence Process Evaluation [Dataset] (2024), 10.5523/bris.3vj9nmc9bekm62pvlyi452jlv2.

